# Artificial Intelligence in Resuscitation: A Scoping Review

**DOI:** 10.3390/jcm12062254

**Published:** 2023-03-14

**Authors:** Dmitriy Viderman, Yerkin G. Abdildin, Kamila Batkuldinova, Rafael Badenes, Federico Bilotta

**Affiliations:** 1 Department of Surgery, Nazarbayev University School of Medicine (NUSOM), Kerei, Zhanibek khandar Str. 5/1, Astana 010000, Kazakhstan; drviderman@gmail.com; 2 Department of Mechanical and Aerospace Engineering, School of Engineering and Digital Sciences, Nazarbayev University, 53 Kabanbay Batyr Ave., Astana 010000, Kazakhstan; 3 Department of Anaesthesiology and Intensive Care, Hospital Clìnico Universitario de Valencia, University of Valencia, 46001 Valencia, Spain; 4 Department of Anesthesia and Intensive Care, University La Sapienza, 00185 Rome, Italy

**Keywords:** cardiac arrest, premature mortality, artificial intelligence, resuscitation

## Abstract

Introduction: Cardiac arrest is a significant cause of premature mortality and severe disability. Despite the death rate steadily decreasing over the previous decade, only 22% of survivors achieve good clinical status and only 25% of patients survive until their discharge from the hospital. The objective of this scoping review was to review relevant AI modalities and the main potential applications of AI in resuscitation. Methods: We conducted the literature search for related studies in PubMed, EMBASE, and Google Scholar. We included peer-reviewed publications and articles in the press, pooling and characterizing the data by their model types, goals, and benefits. Results: After identifying 268 original studies, we chose 59 original studies (reporting 1,817,419 patients) to include in the qualitative synthesis. AI-based methods appear to be superior to traditional methods in achieving high-level performance. Conclusion: AI might be useful in predicting cardiac arrest, heart rhythm disorders, and post-cardiac arrest outcomes, as well as in the delivery of drone-delivered defibrillators and notification of dispatchers. AI-powered technologies could be valuable assistants to continuously track patient conditions. Healthcare professionals should assist in the research and development of AI-powered technologies as well as their implementation into clinical practice.

## 1. Introduction

Cardiac arrest is associated with significant premature mortality and disability worldwide [1,2]. In 2017, in-hospital cardiac arrest (IHCA) occurred at a rate of 8.27 per 1000 hospitalizations and 1.56 per 1000 hospital stays [1]. Despite the fact that the death rate has been steadily dropping over the previous decade, approximately 25% of patients survive until their discharge from the hospital and only 22% of survivors achieve good clinical status [1]. The management of patients with cardiac arrest is associated with several major healthcare issues:

- Cardiac arrest is followed by a short period during which the patient can be resuscitated; if the arrest is not immediately noticed, the chances for successful resuscitation drop rapidly. Unfortunately, the recognition of cardiac arrest and initiation of cardiopulmonary resuscitation are frequently delayed, leading to poor outcomes.
- Survivors of cardiac arrest frequently develop significant neurological problems and even experience post-anoxic coma as part of their poor and unpredictable long-term outcomes. Critical care has made significant advances in recent decades and modern organ-replacing technologies can support life for an extended time. However, this can lead to an increase in the number of patients in vegetative states.
- Cardiac arrest can occur anywhere, and defibrillators are often not available.

Artificial intelligence (AI) could potentially solve these issues. AI could improve the early recognition of cardiac arrest and potentially even predict cardiac arrest or life-threatening arrhythmia through continuous ECG monitoring. AI could also improve the prediction of the outcomes of cardiac arrest, including neurological outcomes.

Traditional prediction methods have poor sensitivity and specificity. To achieve a high level of accuracy, the prediction model should include a large number of pre-arrest variables as well as clinically important parameters linked with cardiac arrest, ensuring optimal prognosis. This proof prediction of the likelihood of good neurological results may offer significant treatment advantages over current methods. Traditional forecasting algorithms for estimating the neurological consequences of IHCA are derived from traditional statistical approaches and grading techniques; these algorithms have shown somewhat adequate prediction capabilities [3,4,5]. An artificial neural network (ANN) is a guided computational model that may incorporate a wide range of dependent and independent variables through continuous training and confirmation by utilizing information to effectively predict the outcome of an unknown situation. ANNs have been used in a number of diagnostic imaging and decision-making applications [6,7]. The objective of this scoping review was to review relevant AI modalities and main potential applications of AI in resuscitation.

We aimed to answer the following questions:

- To identify and characterize the AI models used for the prediction of cardiac arrests and heart rhythm disorders and the development of early warning system;
- To characterize the AI models used for the development of AI-based dispatch rules for drone-delivered defibrillators and the notification of dispatchers;
- To characterized AI-models used for outcome prediction.

## 2. Methods

### 2.1. Protocol

This scoping review protocol was developed, written, and approved by each author. We followed the Preferred Reporting Items for Systematic Reviews and Meta-Analyses (PRISMA) guidelines for scoping reviews [8]. This scoping review is mainly focused on studies presenting the implementation of AI in resuscitation.

### 2.2. Literature Search

PubMed, EMBASE, and Google Scholar databases were utilized to find relevant studies. We used the search terms and their combinations.

### 2.3. Eligibility Criteria

We considered articles reporting the application of AI methods in resuscitation, including early warning and prediction of cardiac arrest, life-threatening heart rhythm disorders, outcomes of cardiac arrest (e.g., post cardiac arrest anoxic coma, delivery of defibrillators by drones). There were no restrictions based on age, gender, geographical region, or type of AI algorithm. We included peer-reviewed publications, articles in the press, written in English. We excluded study protocols, review articles, and conference abstracts. No restrictions were set on the study setting, patient population, study design, country, or time period of the publication year. 

### 2.4. Search Terms and Their Combinations

(((cardiac arrest) OR (tachycardia, ventricular)) AND (artificial intelligence)) OR (machine learning); (“heart arrest” OR (“heart” AND “arrest”) OR “heart arrest” OR (“cardiac” AND “arrest”) OR “cardiac arrest” OR (“tachycardia, ventricular” OR (“tachycardia” AND “ventricular”) OR “ventricular tachycardia” OR (“tachycardia” AND “ventricular” [All Fields]) OR “tachycardia ventricular”) AND (“artificial intelligence” OR (“artificial” AND “intelligence”) OR “artificial intelligence”)) OR (“machine learning” OR (“machine” AND “learning”) OR “machine learning”).

Articles that did not match the review title and abstract sample selection were omitted. The following information was compiled: author, year, mean age, sample size, age, outcomes, AI algorithm, study goals (primary and secondary), sample size (i.e., how many patients were enrolled in the study), diagnosis, comorbidities, AI algorithm/model or method, goals of AI application, advantages, disadvantages, sensitivity, specificity, positive predictive value, and negative predictive value. 

The obtained data were extracted and organized into the following tables: study and cohort information (Table S1), AI methods and characteristics (Table S2).

### 2.5. Data Extraction

Three reviewers independently extracted the data according to the protocol. The data were arranged into three Excel tables. Any conflict between the reviewers was resolved by achieving a consensus among all authors. We performed a narrative synthesis of the extracted data. The findings from the included studies were categorized into the following subsections: article characteristics, goals of the studies, AI models used, and reported benefits of using AI in cardiac arrest patients.

## 3. Results

### 3.1. Study Selection

**Figure 1:**
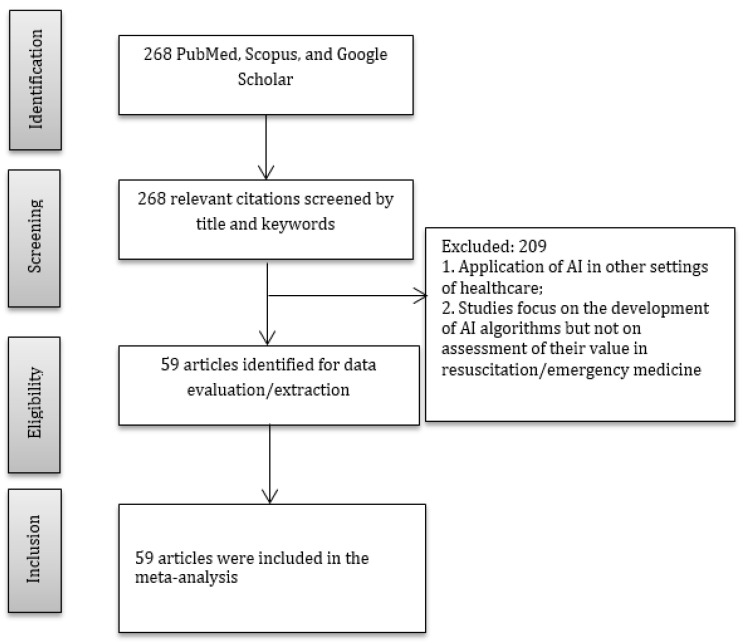
PRISMA diagram.

The systematic search identified 268 original studies, from which 59 original studies (reporting 1,817,419 patients) were chosen for the qualitative synthesis (Figure 1, Table S1). 

### 3.2. Prediction Methods and Goals

The majority of included studies focused on the development of prediction models [9,10,11,12,13,14,15,16,17,18,19,20,21,22,23,24,25,26,27,28,29,30,31,32,33,34,35,36,37,38,39,40,41,42,43,44,45,46,47,48,49,50,51,52,53,54,55,56,57,58,59,60,61,62,63,64,65,66,67]. The main goals of the studies are listed below:

(a) detection of cardiac arrest; (b) development of AI-based early warning system for the prediction of IHCA, OHCA, and life-threatening dysrhythmia; (c) prediction of success of defibrillation and prediction of early outcomes of cardiac arrest; and (d) neurological outcome prediction in comatose patients after cardio-pulmonary resuscitation (CPR).

### 3.3. Prediction of Cardiac Arrests and Heart Rhythm Disorders and the Development of Early Warning System

A wide variety of studies have tried to predict cardiac arrests and mortality in critically ill patients (i.e., those suffering from both IHCAs and OHCAs) using machine learning (ML). Most studies used databases with continuous monitoring of vital and physiological functions, diagnostic tests (e.g., laboratory work), and imaging techniques.

Lee et al. aimed at validating DEWS in a large, multiple-center cohort and comparison of DEWS and modified early warning score (MEWS) in terms of the predictive performance of IHCA [9]. Authors compared DEWS and MEWS using the data collected at several medical centers. DEWS forecasted IHCA better than MEWS and minimized the proportion of false alarms. The results showed that DEWS can be useful in the rapid identification of high-risk individuals. DEWS can forecast and identify patients at risk of IHCA, allowing healthcare professionals to better treat early signs and symptoms [9].

The next groups of studies focused on the development of a tachycardia onset prediction network based on deep learning (i.e., bidirectional long/short-term memory) for early tachycardia detection (Table S1) [11,17,46]. Liu et al. developed a TOP-Net for actual assessment and forecasting of the probability of tachycardia, making it feasible to foresee tachycardia six hours before the event. TOP-Net was evaluated using six metrics, three sub-experiments, and various forecasting periods between zero and six hours. TOP-Net demonstrated better performance compared to the other five existing techniques, including deep learning algorithms, ensemble algorithms, and ANN. The algorithm that included individual details from systems performed better than those that did not. The model’s widely obtainable input data and strong performance in the hospital suggested that early tachycardia development prediction using portable sensing devices could be viable in medical facilities or homes [11].

One study tested the application of machine learning in predicting cardiac arrest based on the combination of imaging and clinical data. Thus, the risk of cardiac death was predicted based on the findings of clinical data and myocardial perfusion SPECT, improving the prediction accuracy and reducing the number of input variables [50]. It was shown that a support vector machine algorithm was the most accurate algorithm, though it required a large number of features, whereas a LASSO model achieves high accuracy with only six variables.

### 3.4. Development of ML-Based Dispatch Rules for Drone-Delivered Defibrillators and the Notification of Dispatchers

The next group of studies investigated the application of AI in notifying dispatchers, as well as in creating and testing machine learning-based dispatch rules for drone-delivered defibrillators. 

More specifically, the published studies focused on the following problems in resuscitation and emergency care:

- Creating and testing machine learning-based dispatch rules for drone-delivered defibrillators using supervised learning models. The ambulance response time was predicted, and these predictions were utilized to determine whether to deploy drones based on an estimated drone flight time.
- Investigation of how OHCA recognition could be impacted by a machine learning model trained to detect OHCA and notify dispatchers during emergency calls [14].

The authors developed dispatch rules for drones carrying automatic electronic defibrillators to the area of suspected OHCA, with the goal of drones reaching the zone of the incident before an ambulance [13,14]. The theory was motivated by the fact that OHCA occurring in remote and rural areas is associated with lower rates of survival and timely access to automated external defibrillators posing a significant challenge [13]. It was found that drone-delivered automatic defibrillators can significantly shorten the time required for help to reach the area of the accident [13].

A similar study was aimed at examining whether the ML model improved medical dispatching and shortened the time-to-recognition of OHCA and the time until dispatcher-assisted cardiopulmonary resuscitation began. The motivation was to use the ML-augmented dispatching to increase recognition of OHCA [14]. The authors did not find improvement in recognition of OHCA supported by ML, although AI surpassed humans in recognizing cardiac arrests [14].

### 3.5. AI in Outcome Prediction

The group of studies focused on the prediction of long-term outcomes (including neurological outcomes) reported valuable results. The studies targeted the following goals:

- Creating and verifying “a machine learning-based outcome prediction model for out-of-hospital cardiac arrest” with an initial shockable rhythm that could be employed when the patient arrives at the hospital [15];
- Identifying the combinations of heart rate variability and heart print indices that can predict sudden cardiac death with a support vector machine using short-term recordings [17];
- Developing a model for predicting outcomes with artificial neural network. (Secondary: using the model to investigate the impact on “illness severity in patients treated with targeted temperature management”) [18];
- Predicting outcomes of comatose patients after CPR to assess the potential contribution of functional magnetic resonance imaging (RS-fMRI) to predict neurological outcomes [19];
- Developing “an accurate and reliable model to predict neurological outcomes in patients with IHCA” based on pre-resuscitation characteristics and identifying the critical variables that contribute to neurological outcomes using ANNs [10].

Thus, Chung et al. found the most significant predictive factors in IHCA-resuscitated individuals and constructed ANN algorithms that can accurately and reliably determine dynamic neurological consequences [10]. As a result, novel machine learning-based algorithms for generating new data and improving medical decision-making can be developed. In particular, the suggested algorithms have the potential to be used in exigent medical circumstances of IHCA, where they can help with the selection and construction of tailored post-resuscitation measures. These models can also be valuable in supporting decision-making processes for optimal management in the post-resuscitation period [10]. 

Andersson et al. developed an ANN that showed high predictability of neurological prognosis in comatose patients after OHCA, using diagnostic biomarkers collected during the first three days of treatment in the hospital [12]. The models, which incorporated neuron-specific enolase (NSE) after 72 h and neurofilament light (NFL) every day, performed well in terms of prognostication. Using purely clinical data, the AUROC remained below 90% during the first three days in the ICU. AUROC increased from 82% to 94% (p 0.01) when clinically accessible indicators such as NSE were included. After adding study biomarkers, the prognosis accuracy was outstanding from Days 1–3, with an AUROC of over 95%. The models that incorporated NSE after 72 h and daily NFL had minimal false positive rate forecasts on each of the three days, as well as a minimal rate of false-negative forecasts [12].

The severity of brain injury is the central determinant of outcomes in patients who sustained cardiac arrests [22]. The multimodality monitoring and neurological evaluation appears to be the main principles of neurological prognostication. The authors investigated patterns of early post-cardiac arrest brain injury using several diagnostic methods and found distinct patterns of post-cardiac arrest neurological injury that could be detected on patient presentation. These patterns indicate that there might be important inter-individual heterogeneity that cannot be easily captured by a standard clinical examination of comatose patients. Outcomes varied widely across clusters. Therefore, authors did not suggest using that AI model for decision-making regarding life-sustaining therapy. Nevertheless, the initial patterns of neurological injury could be useful in personalizing resuscitative actions (e.g., hemodynamic targets or targeted temperature management) [22].

Kwon et al. presented the values for the validation data of 8145 subjects. The logistic regression (for both neurological recovery and survival to discharge) was taken for the control indicator as the best-performing machine learning model [41].

### 3.6. AI Models Used in the Included Studies

The AI models used in the studies are deep learning-based early warning score (DEWS), TOP-Net, long/short-term memory (LSTM), BiLSTMTTM, linear regression and neural network, emergency medical services machine learning-based prognostic model, DLA with CNN, support vector machines, random forest, K-prototypes clusterization, stacking algorithm of support vector machines, decision tree, logistic regression, KNN, GaussianNB, CNNs-Grad-CAMCNN with a VGG, multilayer perceptron (MLP), deep learning-based prognostic system (DCAPS), embedded fully convolutional network (EFCN), GBM, SVC, ensemble feature space embedding, time series forecasting, least absolute shrinkage and selection operator (LASSO), k-nearest neighbor (k-NN), and multilayer perceptron neural network (Table S2) [9,10,11,12,13,14,15,16,17,18,19,20,21,22,23,24,25,26,27,28,29,30,31,32,33,34,35,36,37,38,39,40,41,42,43,44,45,46,47,48,49,50,51,52,53,54,55,56,57,58,59,60,61,62,63,64,65,66,67]. 

### 3.7. The Studies Reported the following Benefits of the Using AI in Cardiac Arrest Patients

- Performance improvement in forecasting IHCA and evaluating deteriorating patients [9];
- Forecasting with fewer alarms and earlier predictions of IHCA. Moreover, AI can be used globally with no technological limitations [9];
- Recognition of complicated nonlinear correlations between dependent and independent variables, and the ability to distinguish all conceivable interactions among predictor variables [10];
- AI models such as TOP-Net can forecast heart rhythm disorder up to six hours in advance [11];
- ANNs predict neurological prognosis in comatose patients after OHCA using clinical factors and biomarkers from the first three days of intensive care, with good-to-excellent predictive accuracy [12];
- Ability to dispatch a drone to a suspected OHCA based on the forecast that it will arrive at the event before an ambulance, which can achieve a comparable distribution of first response times through a policy that dispatches a drone to all OHCAs. Drone-delivered AEDs may dramatically minimize the time required for AED arrival on-site [13];
- Automatic prediction of poor prognosis in OHCA patients with an initial shockable rhythm [15];
- Predicting cardiac arrest using ECG, with a high NPV percentage (over 99%) and the possibility to predict cardiac arrest using single-lead ECG. This method can predict cardiac arrest using wearable devices [16];
- Deep learning-based early warning system can detect patient deterioration earlier according to the vital signs and laboratory results, preventing deterioration [25];
- Good predictive ability of both good and poor outcomes of coma. The study improved the understanding of changes in the brain at a post-anoxic comatose state [27];

Machine-learning models (random forest classifiers) utilizing quantitative EEG reactivity data can predict long-term outcomes after cardiac arrest [30].

## 4. Discussion

Over past several years, several models for predicting cardiac arrest and/or disorders of heart rhythm (especially early risk prediction of patient condition deterioration or adverse events) have been designed based on physiological and vital sign monitoring or data from electronic health records. This scoping review presents an overview of the main directions and perspectives of the application of AI in the management of cardiac arrests and life-threatening heart rhythm disorders. We summarized the main characteristics, diagnosis, comorbidities, AI algorithms, and advantages and disadvantages associated with using the developed AI models.

The selected studies focused on the detection and prediction of cardiac arrest, heart rhythm disorders, and cardiac arrest outcomes (after successful CPR), as well as the notification of dispatchers and the development and testing of machine learning-based dispatch rules for drone-delivered defibrillators and AI-based early warning systems. The selected studies were heterogeneous in quality in terms of design, settings, patient population, causes of cardiac arrest, and AI algorithms. However, all of them have clinical value and might pave the way for the further development of new avenues in AI-assisted resuscitation research. 

Thus, Shamout et al. [28] developed a deep interpretable early warning system that achieved impressive sensitivity of 100% and PPV of 100% in the detection of the deterioration of patient condition. They utilized an attention-based neural network learning algorithm using historical trends of physiological data (vital signs) through mean (interpolated) and variance features to monitor the deterioration of a patient’s condition. The DEWS architecture achieved a high level of performance, even when using a limited set of features. DEWS reduced the number of triggers compared to NEWS, particularly in younger patients. Reducing the rate of false alerts can reduce the burden on medical professionals working in a highly stressful environment. The authors reported that the model achieved the highest level of performance in three individual outcomes (i.e., cardiac arrest, unplanned ICU admission, and mortality) and the composite outcomes. The authors demystified the deep learning model’s decision-making process by adjusting the attention weights corresponding to each vital sign. The trend analysis could be used by physicians to make a decision on appropriate interventions [28].

Another impressive achievement was reported by Tjepkema-Cloostermans et al., who achieved a PPV of up to 100% in predicting poor neurological outcomes in post-anoxic coma patients [36]. They developed a classifier for neurological outcome prediction that provided rapid and reliable forecasts and could be used bedside. The classifier used a convolutional neural network for the extraction of the EEG patterns [36].

### 4.1. Improving Model Performance and Reducing the False Alarm Rate

The true and false alarm rate is an important parameter for validating the applicability of the early warning system because too-sensitive alarms with excessive rates of false alarms can cause alarm fatigue, leading to staff desensitization and failures to respond to clinically significant alarms, putting patient care and safety at risk [68,69]. Thus, an ideal warning system should be highly sensitive while, at the same time, having a low false alarm rate. The modified early warning system demonstrated variable accuracy. Therefore, it was not a sufficiently appropriate rapid response system [70,71].

DEWS was developed in 2018 based on four basic vital signs: systolic blood pressure, heart rate, respiratory rate, and body temperature [41]. Lee et al. expanded the initial version of DEWS by adding age, diastolic blood pressure, and the recorded time of each vital sign [9].

### 4.2. Importance of Timeliness (Trying to Achieve Earlier Prediction at the Same Specificity Level)

A delayed response “is associated with a poor outcome” [72]. Predictable IHCA is defined as “CA that occurs in hospitalized ward patients who met the hospital’s escalation threshold at least 30 min prior to and within 24 h of the event” [73]. The period between 24 h and 30 min prior to IHCA is the appropriate time for a rapid response system to prevent an event [73]. It is important that the staff is aware of the at-risk patients as early as possible, so they have enough time to prepare and to plan action before the event. It is especially important in the general wards, where the patients’ vital parameters are not monitored continuously compared to the continuous monitoring in ICUs [9]. Numerous studies have tried to predict mortality in ICU patients using machine learning (ML) [74,75,76]. Most of the AI- or ML-based studies focusing on mortality or critical event prediction, such as hypotension or sepsis, “achieved better performance compared to conventional prognostic systems” [77,78].

ICUs produce a massive amount of data consisting of a continuous stream of vital parameters, laboratory tests, imaging, microbiological data, fluids, drugs, and transfusions. AI-based (especially ML) prediction methods are much superior to traditional “static” methods in achieving high-level performance if these massive data sets are available [79]. 

### 4.3. Strengths and Limitations

The scope of this review included all applications of AI in the management of cardiac arrest and life-threatening arrhythmia, and several AI modalities could potentially improve the survival and quality of care for patients suffering from cardiac arrest. 

While we performed a systematic literature search using several databases, nevertheless, some relevant articles might have been missed. Another limitation is that the scope of this review was focused on all applications of AI in the management of cardiac arrest; in some studies, it was impossible to eliminate the investigator’s bias in data collection and analysis. Some medications, such as beta-blockers, might influence the study results. The included studies focused on heterogeneous patient populations. 

There was also a risk of missing and incorrect data used for algorithm development. As in many other studies related to AI, there was a chance of overfitting and worse performance in different datasets.

AI in medicine is an emerging field and the number of studies is rapidly expanding. It is challenging to synthesize the information because of the heterogeneous reporting formats and study designs, as well as slight differences in definitions. Likewise, in many other studies, there was a lack of transparency regarding the algorithms and models; therefore, it was difficult to assess the study methodology. Another limitation was that we only included studies published in scientific journals and did not include conference papers or papers published in archives. 

While AI appears to be a valuable option for predicting many life-threatening conditions, the current evidence still has some uncertainties. The majority of studies were based on retrospectively collected data sets. Therefore, there is a need for further validation in prospective and even controlled clinical trials. Retrospective evidence might not be enough to integrate AI into clinical practice safely, especially considering the risks AI poses to results in bias via underfitting or overfitting. Therefore, prospective clinical studies are essential to minimize the risks of biases that might confound results.

Although there is optimism regarding the power of AI in diagnosis and prediction, as well as the transformation of healthcare, there is a long way to go before these algorithms can be reliably used in clinical settings. Even in radiology and medical imaging, a field where numerous promising ideas about outperforming medical professionals are reported, there is still a lot of work required.

The major obstacle for the application of AI in resuscitation is related to the reliability and reproducibility of algorithm results. Although numerous studies reported outstanding results, the use of AI has still mostly limited to research purposes and not yet widely implemented or used in real clinical practice. Before implementation, these models should ideally be validated or tested on the local populations where those models are intended to be used. 

### 4.4. Future Directions

AI might be valuable in the analysis and interpretation of vital sign monitoring and electronic health records, leading to the identification of the most sensitive and specific parameters for predicting life-threatening arrhythmia, cardiac arrest, and the outcomes of cardiac arrest. With the increasing adoption of the internet of things, including wearable devices equipped with sensors tracking physiological functions, AI-powered technologies are now valuable assistants that can track patient conditions around the clock. Healthcare professionals should assist in the research and development of AI-powered technologies as well as their implementation into clinical practice. In order to develop algorithms suitable for a larger patient population, there is a need to include heterogenous training datasets.

## 5. Conclusions

AI might be useful in the prediction of in-hospital and out-of-hospital cardiac arrest, heart rhythm disorders, and neurological outcomes after in-hospital cardiac arrest, as well as aiding in the delivery of drone-delivered defibrillators and the notification of dispatchers. Future prospective studies are warranted to establish more solid evidence in each of the listed applications of artificial intelligence in cardiac arrest management.

## Data Availability

The data will be shared on request to the corresponding author.

## Supplementary Materials

The following supporting information can be downloaded at: https://www.mdpi.com/article/10.3390/jcm12062254/s1, Table S1: Study and cohort information; Table S2: Artificial intelligence method and its purpose.
