# Supplementary material for: Artificial Intelligence in Resuscitation: A Scoping Review
Source: J Clin Med. 2023 Mar 14;12(6):2254. doi: 10.3390/jcm12062254 (PMC10054374; doi:10.3390/jcm12062254)
Supplement: Supplementary file 1 [file jcm-12-02254-s001.zip › jcm-2217821-supplementary file.pdf]

Table S1. Study and cohort information

№	Author, country, year	Study type	Study goals (primary, secondary)	Diagnosis
1	Lee et.al. Republic of Korea, 2021 [9]	Retrospective cohort study	Compare prediction, alerting, and promptness output with the modified early warning score (MEWS)	IHCA
2	Chung et.al. Taiwan, 2021 [10]	Retrospective study	ANNs to develop a reliable model for prediction of neurological outcomes; to identify the critical variables for neurological outcomes	IHCA
3	Liu et.al, China, USA, 2021[11]	Randomized, post-hoc analysis	To create a prediction for onset of deep tachycardia (TOP-Net)	Tachycardia
4	Andersson et.al, Europe, Australia, 2021 [12]	Observational retrospective study	To enhance prognostic performance by simulating the complex network of linked neurons and detecting routes and connections of factors and outcomes	OHCA
5	Chu et.al, Canada, 2021 [13]	Prospective cohort study	Creating and testing machine learning-based dispatch rules for defibrillators delivered by drones. Prediction of ambulance response time. The efficacy of restrictions and identify their trade-offs	OHCA
6	Bloomberg et.al., Denmark, 2021 [14]	Randomized controlled trial	To investigate how OHCA recognition and reaction were impacted by a machine learning model and notify dispatchers in the cases of emergency	OHCA
7	Hirano et.al, Japan, 2020 [15]	Retrospective cohort study	The aim was to create and verify an outcome prediction model	OHCA
8	Kwon et al., South Korea, 2020 [16]	Case-control study	Primary: check the hypothesis that DLAs can predict cardiac arrests	Cardiac arrest
9	Martinez-Alanis et al., Mexico, 2020 [17]	Case-control study	Primary: identify the combinations of HRV and heart print indices to predict SCD through SVM by short-term recordings	Sudden cardiac death
10	Johnsson et al., Sweden, 2020 [18]	Cohort study	Primary: developing an outcome prediction model<br>Secondary: using the model to observe the impact on illness severity	OHCA
11	Wagner et al., Switzerland, 2020 [19]	Cohort study	Primary: predict an outcome of comatose patients after CPR	Cardiac arrest
12	Al-Dury et al., Sweden, 2020 [20]	Cohort study	Primary: investigate the importance of different factors in OHCA	OHCA
13	Liu et al., Singapore, 2020 [21]	Case-control study	Primary: develop a machine learning algorithm to predict ROSC and compare it to RACA score	OHCA
14	Elmer et al., USA, 2020 [22]	Cohort study	Primary: identifying various phenotypes of post-arrest brain injury using unsupervised learning	Post-arrest brain injury
15	Arnold et al., USA, 2019 [23]	Prospective cohort study	To compare early warning systems (EWS)	Adult general medicine ward (condition A and C, ICU transfer)

16	Chen et al., Taiwan, 2020 [24]	Retrospective cohort study	To develop the disposition prediction model and compare it with other 3 methods	all non-trauma adult emergency department visits
17	Cho et al., Korea, 2020 [25]	Retrospective cohort study	To compare the early warning system based on AI with the other methods	General ward patients
18	Fernandes et al., USA, 2020 [26]	Cohort study	To build a model for composite critical outcome risk prediction	emergency department patients
19	Pugin et al., Geneva, 2020 [27]	Prospective cohort study	To identify whether MRI can provide the prognostic information	patients comatose following CA
20	Shamout et al. 2019 [28]	Observational study	Primary: proposition of attention based neural network. Secondary: Evaluation of the benefit of Gaussian Process Regression (GPR) model to compare with Carry Forward (CF) and Linear Interpolation (LI)	ICU, Cardiac arrest
21	Kim et al. 2019 [29]	-	To develop Feasible Artificial intelligence by using the Simple Trajectories for Predicting Adverse Catastrophic Events (FAST-PACE) for the prediction of any adverse events	Cardiac arrest, Acute respiratory failure
22	Amorim et al. 2019 [30]	Retrospective observational study	Primary aim: to investigate if QEEG reactivity can predict good long-term outcomes	Cardiac arrest
23	Javan etl al. 2019 [31]	Retrospective cohort study	To predict the cardiac arrest time for intervening and preventing its onset	Cardiac arrest
24	Merath et al. 2019 [32]	Observational studies	Surgical resection complications after hepatopancreatic and colorectal surgery	colorectal, liver, and pancreatic cancers
25	Park Ho et al 2019 [33]	Cross-sectional study	The aim: to train and check the reliability of predictive models for good neurological recovery	Cardiac arrest
26	Jonas et al. 2019 [34]	Cohort study	Primary: to prediction of clinical outcomes. Secondary: using a visualization algorithm for the EEG features identification	Cardiac arrest
27	Ghassemi et al.,USA, 2019 [35]	Retrospective observational	Primary: to discover if there is a time dependence on the selection of the quantitative electroencephalogram features<br>Secondary: how this time dependency improves the prognostic predictions.	Comatose patients with acute hypoxic-ischemic encephalopathy.
28	Seki et al.,Japan, 2019 [36]	Cohort study	Primary goal: investigate the predictive model for OHCA with presumed cardiac aetiology	out-of-hospital cardiac arrest with presumed cardiac aetiology
29	Tjepkema-Cloostermanset al., Netherlands, 2019 [37]	Prospective cohort study	Primary goal: to develop CNN that will predict the outcome of comatose patients<br>Secondary: to divide the score of neurological outcome as CPC categories	Consecutive comatose after cardiac arrest
30	Moon et al.,USA, 2019 [38]	Randomized study	Primary goal: to develop NLP algorithm that can automatically extract data from clinical narratives.<br><br>Secondary goal: to analyze the NLP and its efficiency of managing HCM patients.	Hypertrophic cardiomyography (HCM), which is inherited disease.

31	Wu et al., Taiwan, 2019 [39]	Case-control study	To elaborate artificial intelligence approach of forecasting stable non-ST-elevation myocardial infarction (NSTEMI)	NSTEMI, Unstable angina
32	Jang et al., Korea, 2019 [40]	Retrospective study	To elaborate and check designs of artificial neural network (ANN)	Cardiac arrest in emergency departments
33	Kwon et al., Korea, 2019 [41]	Case-control study	To develop and verify a deep-learning based out-of-hospital cardiac arrest prognostic system (DCAPS) to predict the neurologic recovery and survival to discharge	Out-of-hospital cardiac arrest (OHCA)
34	Harford et al., USA (Illinois), 2019 [42]	Cohort study	To develop a machine learning model for neurological outcome prediction for OHCA	OHCA
35	Blomberg et al., Denmark, 2019[43]	Case-control study	1.to use a machine learning framework for better identification of the OHCA<br>2.to use machine learning framework to detect the OHCA faster<br>3.to determine the group of patients biased by trained dispatchers in hospital or machine learning framework	OHCA
36	Coult et al., USA, 2019 [44]	Retrospective cohort investigation	1.to analyze the group of ventricular fibrillation (VF) waveform measures with and without chest compressions;<br>2.to predict functionally-intact survival from cardiac arrest	OHCA
37	Nanayakkara et al, Australia, 2018 [45]	Cohort study	To predict risk of in-hospital mortality in cardiac arrest patients	Cardiac Arrest
38	Au-Yeung et al, USA, 2018 [46]	Case-control study	Development of warning system of ventricular tachyarrhythmia in heart failure patients	Heart Failure
39	Matam et al., UK, 2018 [47]	Cohort study	Prediction of cardiac arrests in a pediatric ICU	Cardiac Arrest
40	Lee et al., South Korea, 2018 [48]	Cohort study	DL in cardiac arrest prediction	Cardiac Arrest
41	Kwon et al., South Korea, 2018 [49]	Retrospective Cohort study	DL in in-hospital cardiac arrest prediction	Cardiac Arrest
42	Alonso, USA, 2018 [50]	RCS with fivefold cross-validation	Prediction accuracy, measured by AUC;<br>Simplicity of the model, described by ML features.	Cardiac Arrest
43	Tjepkema-Cloostermans, Netherlands, 2017 [51]	Observational cohort study	Predict values of cerebral recovery index, by using combination of quantitative electroencephalography measures	Cardiac Arrest
44	Rad, Norway, Sweden UK, 2017 [52]	Retrospective cohort study	Automatic resuscitation rhythm classification using only ECG, for use in the resuscitation data review process	Cardiac Arrest
45	Hu, USA, 2016 [53]	Retrospective cohort study	Identify a prediction model for predictive accuracy increase and false alarm rates decrease	hematologic malignancy
46	Verma, USA, 2016 [54]	Retrospective cohort study	Develop a hybrid model for CAD cases identification at minimal cost	Coronary artery disease
47	He, China, 2016 [55]	Retrospective cohort study	Prediction performance of combination of VF features with additional attributes	Cardiac arrest

48	Kennedy et. al., USA, 2015 [56]	Retrospective cohort study	Create a prediction algorithm for cardiac arrests which could be caused by different reasons	Diseases as medical, surgical, trauma, cancer, and bone marrow and solid organ transplantation
49	Tylman et. al., Poland 2015 [57]	Retrospective cohort study	To develop a system which has a solution based on data of the patient, and use artificial intelligence (AI) algorithms for the prediction of possible complications	No specific diagnosis, but anamnesis: Chest pain, Cardiovascular diseases
50	Wise et al., USA, 2015 [58]	Retrospective cohort study	To develop a model for surgeons at a tertiary care center	Abdominal aortic aneurysm, ruptured. Aortic aneurysm of unspecified site, ruptured
51	Liu et al., Singapor, 2014 [59]	Prospective cohort study	To identify the most important variables for the prediction of potential risks of major adverse cardiac events (MACE)	Anamneses: non-traumatic chest pain
52	Ebrahimzadeh et al., Iran, 2014 [60]	Retrospective cohort study	To create the algorithm which predicts the cardiac arrest	Coronary artery atheroma, hypertrophy, ventricular fibrillation or could be even absent.
53	Howe et al., UK, 2013 [61]	Retrospective cohort study	To identify algorithms for shock success prediction	
54	Ong et al., Singapore, 2012 [62]	Prospective, nonrandomized, observational cohort study	Primary: to evaluate the new model of ML score<br>Secondary: to identify at higher severity faster	Cardiac arrest in critically ill patients
55	Yang et al., Ireland, 2004 [63]	Retrospective observational study	Defibrillation outcomes prediction	Ventricular fibrillation
56	Le Duff et al., France, 2004 [64]	Retrospective analysis of data collected from April 1998 to April 2002	Primary: identify variables that were the most important for patient survival<br><br>Secondary: to produce a real patient profile to determine the best practice for him/her.	Cardiac arrest
57	Chong et al., Taiwan, 2003 [65]	Retrospective cross-sectional study	Primary: identify the ANN model based on preoperative clinical and laboratory data	-
58	Zoni-Berisso et al., Italy, 2001 [66]	Prospective cohort	Primary: to identify both clinical and laboratory variables which predict the occurrence of arrhythmic events	myocardial infarction

59	Turton et al., UK, 2002 [67]	Retrospective study	Primary: to identify the set of prognostic variables which predicts outcome for individual patients accurately	RAAA

Table S2. Artificial intelligence method and its purpose

№	Author, country, year	AI algorithm, model, or method	Benefits of AI	Risks of AI
1	Lee et.al. Republic of Korea, 2021 [9]	DL, DEWS	Better performance in forecasting IHCA; better forecasts; no technological limitations.	
2	Chung et.al. Taiwan, 2021 [10]	ANN	minimal training; to recognize complicated nonlinear correlations; to identify all predictor variables' interactions.	Disparity in the IHCA outcomes does not have enough information on the resuscitation process, unable to eliminate investigator bias in data collection and analysis;
3	Liu et.al, China, USA, 2021[11]	TOP-Net, LSTM BiLSTM	forecasts of 6 hours in advance, TOP-Net beat benchmarks of two models, two ensemble models, and one neural network model.	Minimal data, beta-blocker influence that might underseen
4	Andersson et.al, Europe, Australia, 2021 [12]	TTM trial	change in study cohort and removal of patients	ANN may not be preferable in the presence of very strong biomarkers; restricted number of patients for model construction; EEG, SSEP, or neuroradiological imaging were not performed after admission.
5	Chu et.al, Canada, 2021 [13]	linear regression and neural network (Supplemental Methods), EMS	a drone will arrive at the event before an ambulance; drone-delivered AEDs may significantly minimize the AED time	Weather, drone dependability, routing, or impediments in the flight route; missing and incorrect data
6	Bloomberg et.al., Denmark, 2021 [14]		Early exposure to the machine learning model could teach medical dispatchers; additional training to dispatchers could be offered; the downtime of phone calls.	No significant dispatcher recognition improvement
7	Hirano et.al, Japan, 2020 [15]	machine learning-based prognostic model	machine learning approaches to predict the poor prognosis	Poor generalizability; bias due to missing data on five variables being deleted;
8	Kwon et al., South Korea, 2020 [16]	DLA with CNN	High NPV percentage (over 99%) with possibility to predict cardiac arrest using single-lead ECG.	
9	Martinez-Alanis et al., Mexico, 2020 [17]	SVM	SVM could be used on low number of samples	Chance of overfitting and worse performance on different dataset.

10	Johnsson et al., Sweden, 2020 [18]	ANN	Better performance	Imprecise data collection and missing values. Applying ANN in real life is risky due to complexity of biology and other variable factors.
11	Wagner et al., Switzerland, 2020 [19]	Random forest	Computationally effective, good prediction performance, low sensitivity to noise.	different timings of data in taking MRI scans between survivors and non-survivors.
12	Al-Dury et al., Sweden, 2020 [20]	Random forest	Resistant to overfitting; can work with many predictors; detect non-linear association; raw data can be used	Only 16 predictors were examined; one of the first studies
13	Liu et al., Singapore, 2020 [21]	Random forest	Random forest outperformed RACA	
14	Elmer et al., USA, 2020 [22]	K-prototypes clusterization	Can cluster mixed data types, robust handling of missing data	Each brain injury is specific; generalized developed method. Choosing data with appropriate parameters may bias the results
15	Arnold et al., USA, 2019 [23]	RI (Rothman index) Automated EWS (early warning system)	RI was previously tested and showed good result at identifying patients at high risk	Performance is depended on the quality EHR-documented<br>nursing assessments.
16	Chen et al., Taiwan, 2020 [24]	Deep neural network (DNN) model with word embedding on the bases of physician's clinical narratives	Model based on BiLSTM<br>LSTM and CNN;<br>Triage level improves predictive ability	The model does not take into account the medical history
17	Cho et al., Korea, 2020 [25]	DEWS (deep learning based early warnings system)	DEWS is implemented on the hospital. Method detects the case of deterioration earlier in comparison with others.	There could be different performance of method in other hospitals. The study was retrospective view.
18	Fernandes et al., USA, 2020 [26]	Extreme gradient boosting classifier (XGBoost) including chief complaint	Large sample size makes the accuracy of the model high.	The model does not show a good result in emergent and very urgent patients
19	Pugin et al., Geneva, 2020 [27]	resting-state fMRI with implementation of support vector machine learning	Method works for about 8%-15% of patients	Small sample size; might have a low accuracy
20	Shamout et al. 2019 [28]	Logistic Regression (LR), a single-layer LSTM network, and a single-layer BiLSTM	Historical data analyzed provide beneficial supplementary information	Data used to train is not considered to be reliable as dataset was private; the dataset should be generalized or public.
21	Kim et al. 2019 [29]	LSTM	FAST-PACE improved the overall performance	Cannot work with unexpected situations

22	Amorim et al. 2019 [30]	Random forest classifier	Lowering the limitations such as overfitting and correlations	Clinical, imaging and EEG data have not been chosen from the most predictive of long-term outcome
23	Javan etl al. 2019 [31]	Stacking algorithm of SVM, decision tree, logistic regression, KNN, GaussianNB	Better sensitivity, AUC metrics and f1-score criteria	The model cannot extract manually computed rules; the model is not generalizable
24	Merath et al. 2019 [32]	Decision tree model	Improvement of performance through time passing; better prediction after gaining new information	It is new; therefore it is unfamiliar in healthcare sphere
25	Park Ho et al 2019 [33]	LR, extreme gradient boosting (XGB), SVM, random forest, elastic net (EN), and neural network	Identification of special care patients	Inability of interpreting the data due to the complexity of algorithms
26	Jonas et al. 2019 [34]	Deep learning ⇒ CNNs Grad-CAM	Importance for future deep learning model usage	Small sample size
27	Ghassemi et al.,USA, 2019 [35]	Time-senstitive model (EEG features+clinical) OR Elastic Net regularized sequential logistic regression model	Can integrate the clinical up to date and novel QEEG features; time-sensitive; gathers all information from preceding time intervals; memory of the past predictions and can combine them	Dependent on the uniform data of EEG, otherwise the prediction will be declined.
28	Seki et al.,Japan, 2019 [36]	Random Forest	Random Forest can be used to data of different variable types; the sex of the patients, prehospital treatments of patients and other factors are considered together.	As training and testing data was extracted from one hospital, it is applicability to other hospitals cannot be determined
29	Tjepkema-Cloosterm anset al., Netherlan ds, 2019 [37]	CNN with a VGG Architecture, which was developed by Oxford Visual Geometry Group	Not necessary to extract the feature from EEG, instead CNN will be trained by given data; less time consuming than usual feature extracting and interpretation methods	CNN discovers feature at EEG recordings and assigns them into necessary and not necessary features is not clear; CNN cannot correctly predict the outcome of coma as it might misinterpret the features
30	Moon et al.,USA, 2019 [38]	Natural language processing algorithm	NLP have shown superior sensitivity results for extraction of all 3 risk factors comparing with billing codes as well as survey responses of patients and registry entries of nurses	Only recognizes featurethe selected and reviewed keywords from clinical narratives; NLP was trained and tested only in Mayo Clinic, Minnesota.
31	Wu et al., Taiwan, 2019 [39]	ANN model	1.Model showed high results of specificity, sensitivity and accuracy<br>2. model presented good results for predicting NSTEMI patients<br>3. model can help to avoid additional unnecessary tests, such as ECG	It was not checked in real clinical circumstances
32	Jang et al., Korea, 2019 [40]	ANN models: multilayer perceptron (MLP), long-short-term memory (LSTM),	Demonstrated good performance compared with non-ANN models	Number of cardiac arrest forecasts are unknown

33	Kwon et al., Korea, 2019 [41]	Deep-learning-based prognostic system (DCAPS);	Demonstrated higher performance compared with other models	It was not checked in real clinical circumstances
34	Harford et al., USA (Illinois), 2019 [42]	An Embedded Fully Convolutional Network (EFCN)	EFCN model demonstrated better performance than other 5 methods	The risks were not presented in the article
35	Blomberg et al., Denmark, 2019[43]	A machine learning framework	1.Successful identifying OHCA from the calls<br>2. presented significantly faster performance in identifying OHCA than dispatchers	It was not checked in real clinical performance
36	Coult et al., USA, 2019 [44]	Ventricular fibrillation (VF) waveform measures with chest compressions	Ventricular fibrillation waveform measures have an opportunity to predict functionally-intact survival	
37	Nanayakkara et al, Australia, 2018 [45]	GBM<br>ANN<br>SVC<br>RF	Highly optimize predictive segregation for death rate caused by cardiac arrest	The lack of pre-hospital data and little or no external validation
38	Au-Yeung et al, USA, 2018 [46]	SVM<br>RF	Efective in most cases; could be easily applied	Excessive false alarms
39	Matam et al., UK, 2018 [47]	Feature space embedding.<br>Time series forecasting.	Early identification and prediction of cardiac arrests	Low external validation
40	Lee et al., South Korea, 2018 [48]	DEWS	More accurate on false alarms	Risk of accuracy when there are high number of alarms
41	Kwon et al., South Korea, 2018 [49]	DEWS	High sensitivity and low numbers of false alarms	Errors of sensitivity may lead to increase increased cardiac arrest and death
42	Alonso, USA, 2018 [50]	Least absolute shrinkage and selection operator (LASSO)	Simplest model, least number of features used	No patient-specific explanation capability, no cohort study
43	Tjepkema-Cloostermans, Netherlands, 2017 [51]	A support vector machine (SVM)	Highest prediction accuracy	Too many features for result interpretation, no cohort study
44	Rad, Norway, Sweden	Random forest classifier	Sensitive to detect cerebral ischemia, ability to work with many variables	Limited additional predictive value, no detection of seizures

	UK, 2017 [52]			
45	Hu, USA, 2016 [53]	Nested cross-validation (CV) for asystole rhythm	Classification of resuscitation rhythms is more efficient for minimal feedback from human experts	Cannot annotate databases, difficulty in identification of borderline rhythms
46	Verma, USA, 2016 [54]	neural network/ multilayer perceptron	Higher positive predictive value	Excessive false alarms
47	He, China, 2016 [55]	Hybrid Method	Hybrid method had better predictions	Accuracy should be increased
48	Kennedy et. al., USA, 2015 [56]	random effect model (AMSA+PSI+ΔAMSA)	Prediction accuracy	No data on long term survival
49	Tylman et. al., Poland 2015 [57]	linear regression, decision tree, neural network and support vector machine and their combinations	Increasing the accuracy and decreasing the number of false alarms, which brings to better treatment.	Too high rate of false alarms
50	Wise et al., USA, 2015 [58]	Bayesian network	It could be used at the hospital departments and specific cases, when doctors could not pay enough attention to the patients and continuously monitor their health.	Low-signals; the data for BN is not good enough
51	Liu et al., Singapor, 2014 [59]	ANN	Forecasting the nonlinear relationships of variables and outcomes;	No algorithm could give 100% warranty and 100% accurate result.
52	Ebrahimzadeh et al., Iran, 2014 [60]	Random forest	It can determine the variables, which predicts risks of MACE.	The study was only at one center, therefore, not generalizable; basis because of separate data analysis.
53	Howe et al., UK, 2013 [61]	k-Nearest Neighbor (k-NN) and Multilayer Perceptron Neural Network (MLP)	Ability to distinguish patients with different conditions	The risks are unknown
54	Ong et al., Singapore, 2012 [62]	SVM	More accurate prediction how the treatment would be going, what will optimize the treatment process.	Retrospective cohort study with small amount of data
55	Yang et al., Ireland, 2004 [63]	A ML-based prediction model	Minimized errors; overcomed overfitting	-Diagnosis grouping should be selected thoroughly<br>-External validation of the score is needed
56	Le Duff et al., France, 2004 [64]	Robust heteroscedastic probabilistic neural networks (rhpNNs).	1)Short ECG segment<br>2)rhpNNs model can make a fast classification prediction and results in the least misclassification rate<br>3)Neural networks should show better results compared with the linear methods	1) Data from animal studies is not applicable to people<br>2) Using different material for testing and training will result in ambiguous results
57	Chong et al.,	ANOVA analysis or Chi2 tests or Mann and Whitney tests; A Kaplan Meier	The whole picture from a data in a shorter time could be seen	Without enough data, regularity in the relationships will not be found

	Taiwan, 2003 [65]	method for survival analysis; Hidden Markov model		
58	Zoni-Berisso et al., Italy, 2001 [66]	Artificial Neural Network (ANN) model.	Can evaluate the input data in case of unknown data or large sets of data or noisy data; can define relationships that are not observed by human; independent and flexible in analyzing categorical and continuous variables; can predict nonlinear relationships of independent and dependent variables	"Black box" interpretation of ANNs is risky for decision making in medical support systems
59	Turton et al., UK, 2002 [67]	Artificial neural Network (ANN) based on the method of Madansky	Could identify a subgroup of high-risk patients	

AI – artificial intelligence
ANN – artificial neural network
AUC – area under the curve
CNN – convolutional neural network
CPR – cardiopulmonary resuscitation
DEWS – deep learning (DL)-based early warning score
DL – deep learning
DLA – deep learning-based artificial intelligence algorithm
ECG – electrocardiography
EMS – emergency medical services
HRV – heart rate variability
IHCA – in-hospital cardiac arrest
LR – logistic Regression
LSTM – long short-term memory
MEWS – modified early warning score
ML – machine learning
MRI – magnetic resonance imaging
NPV – negative predictive value
OHCA – out-of-hospital cardiac arrest
PPV – positive predictive value
RAAA – ruptured abdominal aortic aneurysm
RACA – ROSC after cardiac arrest
ROR – return to organized rhythm
ROSC – return of spontaneous circulation
RS-fMRI – resting-state functional magnetic resonance imaging
SCD – sudden cardiac death
SVM – support vector machine
TOP-Net – tachycardia onset prediction
TTM – targeted temperature management
TTS – track and trigger systems
